# Compassion apps for better mental health: qualitative review

**DOI:** 10.1192/bjo.2023.537

**Published:** 2023-08-04

**Authors:** Eva de Krijger, Ernst T. Bohlmeijer, Elbert Geuze, Saskia M. Kelders

**Affiliations:** Brain Research and Innovation Centre, Ministry of Defence, The Netherlands; and Faculty of Behavioural, Management and Social Sciences, Department of Psychology, Health and Technology, University of Twente, The Netherlands; Faculty of Behavioural, Management and Social Sciences, Department of Psychology, Health and Technology, University of Twente, The Netherlands; Brain Research and Innovation Centre, Ministry of Defence, The Netherlands; and Brain Center Rudolf Magnus, Department of Psychiatry, University Medical Center, The Netherlands

**Keywords:** Compassion, mobile applications, mental health, eHealth, digital health

## Abstract

**Background:**

There is increasing empirical evidence for the positive mental health effects of compassion-based interventions. Although numerous smartphone apps offering compassion-based interventions (‘compassion apps’) are now available for the general public, the quality of these apps has not yet been reviewed. A qualitative review of existing compassion apps serves as a crucial first step toward testing the efficacy of these apps, by identifying good-quality compassion apps that might be worth the investment of a scientific trial.

**Aims:**

The current study focuses on reviewing the quality of existing compassion apps.

**Method:**

Existing compassion apps were identified through searches in the Google Play Store and App Store. The 24 included apps were reviewed on their quality by using the Mobile App Rating Scale, and on their consistency with current evidence by comparing them to existing and studied compassion-based interventions.

**Results:**

Of the 24 included apps, eight were identified that met the criteria of being consistent with existing and studied compassion-based interventions, and acceptable to good overall quality. The other 16 apps failed to meet one or both of these criteria.

**Conclusions:**

Good-quality compassion apps are available, but many of the available apps fail to meet certain quality criteria. In particular, many apps failed to offer sufficient relevant and correct information, or failed to offer this information in an entertaining and interesting way. It is recommended that future compassion apps are based on a clear definition of compassion, offer evidence- and theory-based exercises and implement tools for increasing engagement.

Common mental disorders, such as mood, anxiety and substance use disorders, are estimated to affect one in five people each year,^1^ and mental disorders are a leading cause of disability worldwide.^2^ In addition, it has been suggested that there is an even larger group of people without a mental disorder but who experience a low level of mental well-being.^3^ This reduced mental well-being is related to similar problems in functioning as when common mental disorders are present.^3^ To improve mental health, there is a need for interventions that help to improve mental health as a complete state, ensuring the absence of mental disorders and the presence of a high level of mental well-being.^4^

## Mental health and compassion

With regard to improving mental health, a concept that has been receiving increasing attention is compassion.^5–10^ Compassion can be defined as adaptive responding to physical, mental or emotional pain or unease.^9,11^ This includes pain or unease experienced by other people as well as one's own pain or unease.^9^ Thus compassion can be directed at other people in an effort to relieve their pain or unease, but it can also be a way of responding to oneself, and one's own personal pain or discomfort.^9^ Compassion has been defined in multiple ways, and a review performed by Strauss et al in 2016^11^ aims to bring all those definitions together by defining compassion as ‘a cognitive, affective and behavioral process consisting of five elements: (1) recognizing suffering, (2) understanding the universality of suffering in human experience, (3) feeling empathy for the person suffering and connecting with the distress, (4) tolerating uncomfortable feelings aroused in response to the suffering person, so remaining open to and accepting of the person suffering and (5) motivation to act to alleviate suffering’.

There is increasing empirical evidence that demonstrates a significant association between compassion and mental health.^5–10^ On the one hand, compassion has been shown to be negatively related to the occurrence of mental illness^5–7^ and to factors that are related to mental illness, like self-criticism, rumination, neurotic perfectionism and thought suppression.^9^ On the other hand, there is also a growing body of research that shows positive relationships between compassion and mental well-being, in terms of subjective well-being, life satisfaction, social connectedness, emotional intelligence and adaptive emotion regulation strategies.^8–10^

Furthermore, it has been shown that compassion can be enhanced by offering compassion-based interventions, and that these interventions yield significant positive effects on mental health.^12,13^ Compassion-based interventions are often offered in a face-to-face format, with a therapist or trainer delivering the intervention to an individual or a group of people. A review performed by Kirby in 2016 describes six empirically supported interventions that focus on the cultivation of compassion, such as compassion-focused therapy and mindful self-compassion.^14^ These interventions focus on improving mental health through the cultivation of compassion, and all contain exercises that are explicitly focused on cultivating compassion, such as compassion meditations or practicing compassionate responses to suffering in daily life. In addition to these face-to-face formats, a more recent study offered support for the efficacy of a guided self-help compassion intervention,^15^ suggesting that face-to-face contact is not a prerequisite for the effectiveness of compassion-based interventions. From this point of view, huge potential may also lie in the emergence of smartphone applications that offer compassion-based interventions, or ‘compassion apps’.

## Potential of compassion apps

With smartphone ownership rates fast increasing all over the world,^16^ offering compassion-based interventions via smartphone applications has the potential to reach an increasingly large group of people. An important advantage that compassion apps may offer is that they are available to support compassion practice anytime, anywhere. Thereby enabling more frequent practice. This is important because with regard to compassion-based interventions, a greater level of practice has been shown to be associated with a greater level of compassion.^17^ Furthermore, like other mental health apps, compassion apps have the potential to lower financial costs of mental healthcare,^18,19^ and to reach people who want to improve their mental health but do not use in-person mental health services because of barriers to care, such as time, distance, financial costs and stigma.^20^

With regard to the efficacy of mental health apps in general, recent reviews show favourable results for those apps that have been studied with either a pre–post test design or a randomised controlled trial.^21–23^ However, these same reviews identify the issue that these types of studies are rare, and that the majority of available mental health apps therefore seem to lack scientific evidence regarding their efficacy.^21–23^ The review by Donker et al in 2013 identified only eight studies associated with five different apps; at the time, more than 3000 mental health apps were available to download.^21^ With regard to those apps that have not been scientifically studied, many have been shown to offer content that is not in line with existing guidelines or evidence-based practice.^24,25^ Some mental health apps have even been shown to offer advice that might be harmful, such as suggesting the use of alcohol as a solution for stress-based problems.^26^ Furthermore, mental health apps might differ in their overall quality, both in the quality of the content and the quality of the technology used to deliver the content. A mental health app that offers high-quality content that is in line with evidence-based practice, but does this with low-quality technology that negatively affects the app's attractiveness, ease of use or functionality, may have little effect on level of mental health.^27,28^

The existence of low-quality mental health apps that either fail to follow evidence-based practice or are of low technical quality poses risks for the general public.^29^ Especially because it has been suggested that users are not able to discern high-quality apps from lower-quality apps.^25^ Users might rely on an app's description to assess its quality, but it has been shown that description of apps can be misleading, with certain apps claiming to provide effective mental health strategies but actually failing to do so.^30^ Users have furthermore been shown to rely on app ratings in selecting and adopting mental health apps,^31^ but it has been shown that iTunes star ratings, for example, are only moderately related to an objective measure of health app quality.^32^ Risks of using a low-quality mental health app might consist of not gaining any improvement, which in turn might discourage users in seeking help for their mental health issues altogether, or worsening of mental health status because of harmful advice.^29^ It is, therefore, important to gain information on the quality of currently available mental health apps. Such information can also serve a purpose for mental health professionals in determining which apps to use and/or recommend as an addition to the treatments they offer.

Recent studies have focused on reviewing the quality of mindfulness apps.^33–35^ However, to the best of our knowledge, the quality of compassion apps has not yet been reviewed. Therefore, the current study aims to be the first study that focuses on qualitatively reviewing existing compassion apps, with regard to their overall quality and specifically the extent to which they are in line with evidence-based practice.

## Method

### Search strategy

To achieve the objectives of the study, we systematically identified apps in the Android Google Play Store and Apple App Store that claim to focus on improving compassion and/or entail a considerable number of exercises (at least four) that are focused on improving compassion. Searches for apps were performed on 15 January 2022 for the Android Google Play Store and 16 January 2022 for the Apple App Store. For the search in the Google Play Store, the Google Play Store was entered online via a computer, and the search in the App Store was performed on an iPad (2019 seventh generation, iOS 15.7) with the filter set to apps available for iPhone. The search terms ‘compassion’, ‘*compassie*’ (Dutch for compassion) and ‘*mededogen*’ (a Dutch synonym for compassion) were used. It was a deliberated decision to use only these three search terms, instead of adding related constructs such as ‘kindness’ or ‘empathy’, because it was the intention to specifically identify apps that claim to focus on improving compassion. Compassion has been argued to be similar to, but broader than, constructs such as kindness and empathy, in the sense that it encompasses more than just kindness or empathy.^36^

The resulting apps were screened by checking both titles and descriptions on the following inclusion criteria: (a) the app is in either English or Dutch and (b) the description states that the app offers a framework to improve compassion, or (c) compassion is mentioned as one of the topics that can be worked on with the app. This was done by one researcher. Apps that met the first two inclusion criteria were included regardless of the content they offered. Apps that were in English or Dutch and mentioned compassion as a topic that could be worked on in the app, but did not state that it offered a framework to improve compassion, were downloaded and assessed to check their content regarding the following inclusion and exclusion criteria.

- Inclusion criterium: the app contains at least four exercises that are presented as exercises that help to improve compassion.
- Exclusion criterium: the content of the app is presented as content that helps to improve ‘compassion fatigue’ or ‘compassion satisfaction’ instead of compassion.
- Exclusion criterium: (part of) the app is inaccessible, and this makes it impossible to check whether the app meets the inclusion criterium above.

This was done by two independent researchers, and differences in opinion were discussed to reach consensus. In this selection process we did not reference the content of the app to any definition of compassion; apps were included when the app content was presented as content that helps to improve compassion, regardless of the definition of compassion that was offered by the app.

### Measures

The included apps were reviewed with regard to their quality by using the Mobile App Rating Scale (MARS).^32^ The MARS is a 23-item scale for classifying and rating the quality of mobile health apps. The 23 items are subdivided into four objective quality subscales (engagement, functionality, aesthetics and information quality) and one subjective quality subscale. Items are scored on a five-point scale (1, inadequate; 2, poor; 3, acceptable; 4, good; 5, excellent). The MARS is scored by calculating the mean scores for the four objective quality subscales, and the total mean score of these four scales. For the current study, we chose not to score the subjective quality scale. We feel that this scale is less relevant for the current study because it concerns our subjective experience of the apps. The MARS has demonstrated excellent internal consistency (Cronbach's α = 0.90) for the total score, and very high internal consistencies (Cronbach's α = 0.80–0.89, median 0.85) and fair to excellent interrater reliabilities (intraclass correlation coefficient 0.50–0.80, median 0.65) for the subscales.^32^

The MARS includes an item on evidence base (‘has the app been trialed/tested?’), which has to be verified in published scientific literature. This item is part of the information subscale of the MARS. We scored this item based on a search in Google Scholar, with the name of the app followed by ‘app’ as a search term (e.g. Imagine Clarity app). Next, at least the first page of results was screened by checking titles and first sentences of the abstracts for these search terms. When this search led to articles that referred to the app that was searched for, the search was continued until a full page of results did not show any results that referred to the app. Since it has been shown that the majority of available mental health apps have not been scientifically tested,^21–23^ the included apps were also rated with regard to the extent that they are consistent with existing and studied compassion-based interventions. This was done separately from the scoring of the MARS, using the coding scheme shown in Table 1. The coding scheme was based on a review by Kirby in 2016, in which compassion-focused therapy, mindful self-compassion, compassion cultivation training, cognitively based compassion training and cultivating emotional balance were identified as existing and studied compassion-based interventions.^14^

**Table 1:** Coding scheme for consistency with the evidence base

Criterium for consistency with evidence base	Classification	Coding
The content of the app is consistent with the content of existing and studied compassion-based interventions, in the sense that it is consistent with the concept of compassion as being a way of responding to suffering, by turning the user's attention toward it instead of shutting it out and by responding with kindness, care and/or support	App meets this criterium	Yes
	App does not meet this criterium	No

### Procedure

The included apps from the Google Play Store were reviewed in Android 11 with a Samsung Galaxy A32 5G, and the apps from the App Store were reviewed in iOS 15.4.1 with an iPhone 11. Two independent researchers were involved in reviewing the apps (E.d.K. and S.M.K.). As recommended by the developers of the MARS, both researchers completed a training exercise. This training exercise consists of rating a mental health app, after which the researchers discuss their ratings of this app to reach a mutual understanding of the meaning of the items. Each app was passively tested (i.e. installed without actively opening it) for a minimum of 2 weeks, and actively tested for a minimum of 30 min, by at least one of the two raters. The passive testing time of 2 weeks was taken into account to check the amount and content of reminders/prompts.

A total of 70% of the apps were tested and reviewed on 16 items of the MARS by both researchers. We chose to have part of the apps scored by two researchers to be able to calculate interrater reliability, and this also enabled us to determine certain rules of interpretation for the MARS items based on two different perspectives. An intraclass correlation of 0.82 indicated good interrater reliability. After determining the intraclass correlation, the two researchers discussed the differences in scores and, based on this discussion, determined certain rules of interpretation for the MARS items (included in Supplementary Appendix 1 available at https://doi.org/10.1192/bjo.2023.537). These rules of interpretation were than used by one researcher (E.d.K.) to determine the final scores of the apps, and the scores of the remaining apps. As a consequence, the apps have been scored by one researcher, but these scores are based on the perspectives of two researchers that have both individually studied a large part of the apps extensively.

An exception was made for items 5, 15 and 16 of the MARS and ‘consistency with evidence base’ (Table 2). These items were scored by a single researcher (E.d.K.) for all of the apps. This was decided because to score these items precisely, we had one of the researchers listen to all of the audio and video fragments included in the apps. This researcher worked with specific rules of interpretation for these three items of the MARS (included in Supplementary Appendix 1).

**Table 2:** Results for the Mobile App Rating Scale and consistency with the evidence base

Name of the app	Android/iOS	Engagement	Functionality	Aesthetics	Information	QMS	Consistency with evidence base
The Self Compassion App	Android/iOS	4.8	4.75	4.33	4.29	4.54	Yes
Imagine Clarity	Android/iOS	3.4	4.75	5	4.25	4.35	Yes
Insight Timer – Meditation App	Android/iOS	4.6	4.75	4	3.6	4.24	Yes
Mesmerize – Visual Meditation	Android/iOS	3.8	3.25	5	4	4.01	Yes
Petit BamBou: Meditatie	Android/iOS	4.4	4.75	3.33	3.5	4.00	Yes
Mindshine: Mental Health Coach	Android/iOS	4.2	4.75	3.67	3.4	4.00	Yes
Centre for Mindfulness Studies	Android/iOS	3.2	4.75	3.33	3.75	3.76	Yes
Breathr: Mindful Moments	Android/iOS	3.4	4.75	3.67	3	3.70	Yes
Buddhist Meditations with Vene	Android	1.8	4.5	2.33	3.2	2.96	Yes
Meditopia: Meditatie, Slaap	Android/iOS	4.4	4.5	4	3.2	4.03	No
Samten: Meditation & Sleep	Android/iOS	3.2	4.25	4	3	3.61	No
Piku – Calm Kids	Android/iOS	2.8	5	4	2.6	3.6	No
Humanly: A Mental Health Guide	iOS	3.4	4.75	3.33	2.67	3.54	No
16Guidelines for a Happy Life	Android/iOS	2.4	4.75	4	3	3.54	No
Ommie	iOS	2.2	4.25	4	3.33	3.45	No
Jon Kabat-Zinn Meditations	Android/iOS	3	4.25	3	3	3.31	No
Mindfulness App	Android/iOS	3.4	3.75	3	3	3.29	No
Bodi Posi: Daily Habit	Android	2.4	4.25	3	2.4	3.01	No
3 Good Things daily gratitude	Android/iOS	2.2	4.25	3	2.6	3.01	No
KritterKneads	Android/iOS	2.2	4.5	2	2.67	2.84	No
Yoga For Kids: Kids Fitness	Android	2.2	4.5	2	2.67	2.84	No
Unlock Your Potential – Sleep	iOS	1.8	4.25	2	2.6	2.66	No
Nan Wu Amitabha	Android	1	4.5	2	3	2.62	No
Compassion Today!	iOS	1	3	2.33	3	2.33	No
Median scores of all apps		3	4.5	3.33	3.2	3.54	

Yes indicates that an app is consistent with the evidence base; no indicates that it is not consistent with the evidence base. QMS, quality mean score.

## Results

### Selection of apps for review

**Fig. 1:**
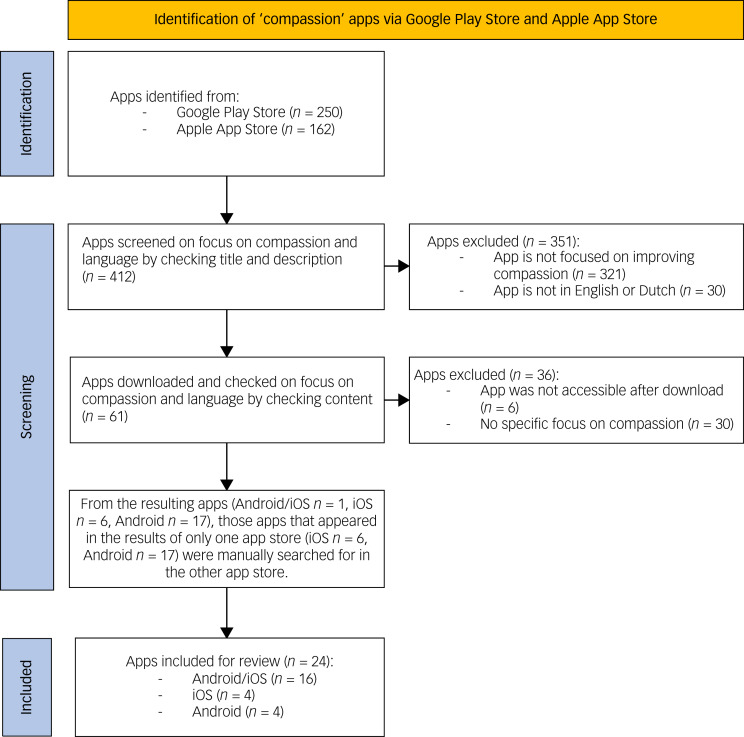
Flowchart of the app screening and selection process.

The search led to a total of 412 apps (Google Play Store *n* = 250, Apple App Store *n* = 162), of which 24 apps could be included. A total of 321 apps were excluded because the title or description did not meet the inclusion criteria with regard to suggesting a focus on improving compassion. These were, for example, apps from religious or charity organisations that used the term compassion in their title but described aims other than improving compassion. A total of 30 apps were excluded after download because although compassion was mentioned in the description as one of the topics that could be worked on with the app, the content did not demonstrate any, or only few, exercises that were presented as exercises that help to improve compassion. These were, for example, apps that offered only one exercise to improve compassion, with the rest of the app focusing on mindfulness or other meditation practices. Of the 24 included apps, one appeared in the search results of both app stores and 23 appeared in the search results of only one of the app stores (either iOS or Android). These 23 apps were manually searched for in the other app store, to check for availability in the other app store. See Fig. 1 for a detailed overview of the selection process. Additional information about the 24 included apps (developer, link, cost, etc.) is included in Supplementary Appendix 2.

### App quality rating

The 24 included apps were reviewed on their quality with the MARS. Table 2 demonstrates the scores on the MARS. The quality mean score of the MARS was calculated as a mean, but in this section, we present the median scores across all apps. The 24 reviewed compassion apps show a median quality mean score of 3.54 (s.d. = 0.58) on a scale ranging from 1 to 5. The apps demonstrated the highest median score for functionality, with a median score of 4.5 (s.d. = 0.47). This reflects that most apps were free of major technical problems and easy to use. The apps demonstrated a median score of 3.1 (s.d. = 1.05) on engagement. A score of 4 or higher on engagement, which is described as good to excellent, was given to only five apps (21%). In fact, 11 apps (46%) had a score that was lower than 3 on engagement, and a score of 3 is described as acceptable by the developers of the MARS.^32^ This reflects the fact 46% of the reviewed apps failed to offer their content in a well-targeted, entertaining and interesting way, with options for customisation and interactivity. The apps demonstrated the lowest median score for information, with a score of 3 (s.d. = 0.51). A score of 4 on information, which is described as ‘good’, was given to only three apps (13%). The 21 apps that received a score lower than 4 varied in the quality of the information that was offered, from not offering any information on compassion at all, to offering good-quality information on compassion but mixing the information with information on many different concepts to the point where the definition of what compassion entails remained hard to grasp.

### Evidence base

None of the 24 apps that were reviewed have been studied in randomised controlled trials. We did find a study protocol for a randomised controlled trial focusing on the effectiveness of the app ‘Insight Timer’ in reducing anxiety and improving well-being during the COVID-19 pandemic, which was estimated to be completed April 2021,^37^ but the results of this trial have not been published yet.

Two apps (8%) have been trialled in studies that are not randomised controlled trials: ‘Insight Timer – Meditation App’^38–40^ and ‘Petit BamBou: Meditatie’.^34^ These studies included three pilot studies in which respectively college students,^38^ military nurse practitioners^39^ and neurological patients^40^ were instructed to use the app ‘Insight Timer’ for a certain period of time. Positive outcomes were reported in terms of significant decreases in state and trait anxiety,^38^ improvement in levels of burn-out and stress in 75% of the participants^39^ and lower pain scores compared with matched controls.^40^ The app ‘Petit BamBou: Meditatie’ was rated by Nunes et al on its quality as one of the available mindfulness apps for children, and it received the highest rating of the apps that were studied.^34^ For each of these studies, the apps were employed or reviewed as ‘mindfulness apps’. These studies did not focus (specifically) on the sections of these apps that included compassion exercises, and compassion was not one of the outcomes that was measured.

### Consistency with evidence base

In addition to checking the evidence base of the apps, we also rated the apps on the extent to which they are consistent with existing and studied compassion based interventions. The results are demonstrated in the last column of Table 2. A total of nine apps received an ‘yes’ on this rating because their content was rated as being consistent with the content of existing and studied compassion based interventions. The remaining 15 apps received a ‘no’ because they failed to meet this criterium. These apps claim to provide a framework for improving compassion in the description of the app, but do so in a way that is not consistent with existing and studied compassion based interventions. In fact, seven apps (29%) failed to even mention the concept of compassion in the app itself, despite the descriptions of these apps stating that they provided frameworks for improving compassion. An example is the app ‘16Guidelines for a Happy Life’, which offers information and exercises to practice with 16 guidelines for life, including ‘kindness’, ‘generosity’ and ‘gratitude’, but compassion is not mentioned anywhere within the app. Another example is the app ‘Yoga For Kids – Kids Fitness’, which states in the description that it ‘fosters cooperation and compassion in your children’, but offers instructions on yoga poses without mentioning compassion anywhere. The other eight apps (33%) did mention compassion, but provided little or no information on what it entails and how to improve it, or misrepresented compassion. Compassion was misrepresented in two ways: (a) by presenting content that was focused on the cultivation of love, kindness and care in general, whereas compassion is defined as love, kindness and care specifically in response to suffering;^11^ and (b) by presenting content that focuses on compassion as the opposite of self-centeredness.

### Top-rated apps

The highest rated app, ‘The Self Compassion App’ (quality mean score of 4.54), is the only app included in this review that solely focused on improving compassion. It contains a step-by-step course that consists of 27 succeeding sessions. Each session is a combination of theory (that is either available as written text or as an audio fragment) and exercise, that help users work with difficult thoughts and emotions with techniques from compassion-focused therapy. The ‘Self Compassion App’ stands out with the high quality of the information that it offers and the engaging way in which this information is offered. It contains different options for customisation and different interactive features (such as a measurement of heart rate variability).

The second-highest rated app, ‘Imagine Clarity’ (quality mean score of 4.35), contains different meditation courses and one of them is presented as a course that is focused on improving compassion. This course is called ‘the dynamics of altruistic love’ and contains a total of seven 20-min meditations. These meditations are focused on building feelings of love, compassion and joy for everyone without partiality. The mediations offer users the experience of simply observing difficult emotions or situations instead of turning their attention away from them, and to allow themselves to be touched by what they observe and look at it from the perspective of wanting all human beings to be happy and free of suffering. Similar to the ‘Self Compassion App’, ‘Imagine Clarity’ stands out with the high quality of the information that it offers. Compared with ‘The Self Compassion App’, ‘Imagine Clarity’ received markedly lower scores for engagement, because it contains little or no options for customisation or interactivity. On the other hand, ‘Imagine Clarity’ received higher scores on ‘aesthetics’, because it offers graphics that score high on attractiveness and a professional-looking layout.

The third-highest rated app, ‘Insight Timer – Meditation App’ (quality mean score of 4.24), contains a great amount of meditation courses and practices, and among these are different compassion practices and different courses that at least touch upon compassion as a subject. The current review was focused on a Dutch course called ‘Making friends with yourself’, which is described as a 10-day course that teaches users to have more compassion for themselves and treat themselves with more kindness, and, as a consequence, experience less stress and be more resilient. Each of the 10 days consists of a meditation practice that focuses either on mindfulness, noticing the good, taking care of oneself, gratitude or the practice of self-compassion. The meditation practice is followed by a multiple choice question that encourages users to reflect on how the material that was presented relates to them, such as ‘Where in your body do you notice what you are feeling most easily?’. ‘Insight Timer – Meditation App’ scores lower on information compared with ‘The Self Compassion App’ and ‘Imagine Clarity’, because the section that was reviewed also focuses on many different concepts (‘noticing the good’, gratitude and forgiveness). These are separate concepts and not part of what compassion entails,^11^ but this is not explained within the app, which might lead to confusion on the definition of compassion. The app does, however, contain a great number of exercises and courses that offer relevant and correct information on what compassion entails and how to improve it. In addition, ‘Insight Timer – Meditation App’ also stands out with the engaging way in which the content is offered by offering numerous options for connecting with other users and teachers. For example, it shows the number of people that are using the app when you are, if you give permission it connects you to colleagues that also use this app and you can join live classes or ‘classrooms’ to ask questions.

## Discussion

Smartphone applications that offer compassion-based interventions, or ‘compassion apps’, are available for the general public, but to the best of our knowledge, these apps have not previously been reviewed on their quality. The current study therefore focused on qualitatively reviewing existing compassion apps. The search for compassion apps led to the inclusion of 24 apps. These 24 apps were reviewed on both the extent to which they are consistent with existing and studied compassion-based interventions, and their overall quality, which included ratings on engagement, functionality, aesthetics and information. Through these ratings, eight compassion apps were identified that, with regard to their content, are consistent with existing and studied compassion-based interventions, and were scored as acceptable to good with regard to their overall quality. Compared with mindfulness apps, which have also been qualitatively reviewed, this score is rather low: a review performed by Mani et al identified 23 mindfulness apps, 20 of which were scored as acceptable to good with regard to their overall quality.^33^

Furthermore, 11 of the 24 apps were scored below acceptable (a score of 3) on the engagement subscale. This result is deemed important because it has been shown that the effectiveness of compassion-based interventions depends on the amount of practice: a greater level of formal compassion practice is associated with higher levels of compassion.^17^ Therefore, less-engaging apps, even when they offer strong or evidence-based content, might be less effective in improving compassion because they might fail to stimulate repeated practice. Engagement has been identified as an issue with regard to digital behaviour change interventions in general, but it has also been shown that it is possible to influence the level of engagement.^28,41^ For example, a review performed by Kelders et al showed that it is possible to use technology to improve adherence with regard to web-based interventions.^28^

Randomised controlled trials with the 24 included apps have not been published in scientific literature to date. Two apps have been trialled in studies that are not randomised controlled trials, and demonstrated positive outcomes in these studies.^34,38–40^ However, these studies did not (specifically) focus on the sections of these apps that included compassion exercises, but rather focused on these apps as mindfulness apps. The scarce scientific evidence base for compassion apps is in line with an earlier review that showed a lack of scientific evidence for mental health apps in general.^21^

In addition to checking the evidence base of the apps, the apps were also reviewed on the extent to which they are consistent with existing and studied compassion-based interventions. These reviews demonstrate that 15 of the 24 available compassion apps claim to provide a framework to improve compassion, but each app does so in a (completely) different way than evidence-based compassion interventions normally do. Some of these apps claim to provide a framework for improving compassion in the description of the app in the app store, but do not even mention compassion within the app itself. These results are in line with studies demonstrating that many mental health apps, **that have not been scientifically studied**, offer content that is not in line with existing guidelines or evidence-based practice.^24,25^ Since the frameworks that these 15 apps offer to improve compassion have not been tested, there is no support that applying the offered exercises will actually lead to an improvement in compassion. The fact that 15 of the 24 available compassion apps offer content that is not in line with evidence-based practice poses a risk for users, as has been suggested for the existence of low-quality mental health apps in general.^29^ This is especially true because users might not be able to discern high-quality from low-quality compassion apps, as has been suggested for mental health apps as a whole.^25^

Another important observation is that from the nine apps that were reviewed as being consistent with evidence-based interventions, six apps were scored below good (a score of 4) on the information subscale. Some apps failed to offer a clear definition of what compassion entails, and other apps mixed the information on compassion with information on many different concepts to the point where the definition of what compassion entails remained hard to grasp. This observation is deemed important because, with regard to face-to-face compassion-based interventions, it has been shown that the effectiveness of these interventions is influenced by the extent to which the recipients understand the concept of compassion.^42^ Therefore, apps that offer sound content, but fail to clearly bring across a definition of compassion, might be less effective.

Given the potential of mental health apps in general, and compassion apps in particular, we would recommend performing scientific trials to test the efficacy of compassion apps. The results of the current review could serve as a first step toward performing scientific trials with compassion apps, by identifying good-quality compassion apps that might be worth the investment of a scientific trial. In the meantime, the results of the current review could serve as a guide for healthcare professionals looking for good-quality compassion apps to recommend to patients. Furthermore, the current review identifies major areas for improvement for the future development of compassion apps: offering interventions that are based on studied frameworks for improving compassion, offering a clear definition of compassion and increasing engagement. Finally, the results of the current review, and the notion that users are unable to tell high-quality apps from low-quality apps,^25^ underlines the importance of a system that supports potential users in judging existing mental health apps on their quality. In this regard, we would like to point to emerging app libraries such as ‘Psyberguide’ and ‘VicHealth’. These online libraries assist users in their selection of a high-quality mental health app, and we believe that it would be beneficial for these libraries to gain publicity among mental health app users.

### Recommendations for health professionals

Interventions that focus on improving compassion help to cultivate a mindful and caring way of responding to pain or difficulty. Instead of either shutting out painful feelings or thoughts or losing oneself in distress over them, people are taught skills to become aware of pain or difficulty and use their innate capacity for love and care to slow down their bodies and feel grounded, and to act in a way that is focused on resolving or relieving suffering. As such, the eight compassion apps in the current review that were identified as meeting the various criteria for sufficient quality (see the top eight apps in Table 2) can be relevant for a wide variety of patients. This includes both those with and without a clinical mental health diagnosis, since compassion interventions have been shown to be relevant both in clinical populations and community samples. These compassion apps may be particularly useful for people that experience high levels of shame or self-criticism, or more generally, people that want to develop a kinder way of relating to themselves, especially at those moments when life gets difficult, such as in situations of failure or loss.

### Limitations

For the current review, the decision was made to include all apps that claim to offer a framework to improve compassion, and to review their quality with the MARS, even when the apps turned out to offer little or no information on the subject of compassion.^32^ The MARS includes ratings on different quality domains (engagement, functionality, aesthetics and information), which are combined to calculate a quality mean score. As a result of this procedure, in which a mean is calculated without giving extra weight to certain quality aspects, combined with our inclusion criteria, certain apps received a high-quality mean score (e.g. ‘16Guidelines for a Happy Life’ and ‘Piku – Calm Kids’) despite not mentioning compassion within the app. We have tried to overcome this limitation by including a rating on consistency with evidence base. We have chosen to base this rating on the extent to which the content of the apps was consistent with existing compassion-based interventions that, according to an earlier review, have been scientifically studied.^14^ However, this review also concludes that the empirical evaluation of compassion-based interventions is still in its infancy, and much more research is needed to test the effectiveness of these interventions. This should be kept in mind with regard to the conclusions of the current review as well. The quality of compassion apps could be rated more precisely if data becomes available on the effectiveness of different individual components of compassion-based interventions.

Finally, it was a considered decision to focus specifically on apps that use the term ‘compassion’ in their description. First, because it was the intention to focus specifically on apps that claim to focus on improving compassion, and compassion has been argued to be similar to but broader than constructs such as kindness and empathy.^36^ Second, this decision was influenced by the fact that, for the Apple App Store, running a search with different search terms is not possible. The search should than be repeated for each search term separately. However, because of this decision, we might have missed compassion apps that describe their content with different terminology. The results and conclusions of this review are therefore limited to compassion apps that use the term ‘compassion’ to describe their content.

In conclusion, compassion for oneself and others has been shown to positively affect mental health in the context of suffering.^5–10^ Compassion apps may be supportive in developing compassion skills and applying compassion in daily life. Based on this review, currently eight of the 24 included apps meet the various criteria for sufficient quality. However, the effects of these apps, or the sections of these apps that contain compassion exercises, have not been studied in trials. So, at present, there is no knowledge about their efficacy with regard to improving compassion. It is therefore highly recommended that future research focuses on evaluating the impact of good-quality apps. In addition, we feel that the field of mobile mental health would profit from further research on what constitutes good-quality apps, or determining that factors that affect the efficacy of mobile mental health apps. Many of the apps did not meet the criteria for sufficient quality that were set for the current review. Many apps failed to offer a clear definition of compassion, or to offer exercises that are evidence and theory based. Furthermore, several apps failed to stimulate repeated use by increasing engagement. It is therefore recommended that compassion apps are developed that offer a clear definition of compassion, offer evidence- and theory-based exercises and implement tools for increasing engagement.

## Data Availability

The data that support the findings of this study are available from the corresponding author, E.d.K., upon reasonable request.

## Supplementary material

For supplementary material accompanying this paper visit http://doi.org/10.1192/bjo.2023.537. click here to view supplementary material
