# Supplementary material for: Compassion apps for better mental health: qualitative review
Source: BJPsych Open. 2023 Aug 4;9(5):e141. doi: 10.1192/bjo.2023.537 (PMC10486246; doi:10.1192/bjo.2023.537)
Supplement: Supplementary file 1 [file bjosup.zip › S2056472423005379sup001.docx]

**Appendix 2**

Rules of interpretation for items of the MARS that were applied in scoring the apps in the current review:

- Rate apps based on what is included in the app itself, instead of also including material online that the app links to.
- Interpret ‘target audience’ in item number five (‘is the app content [visual information, language, design] appropriate for your target audience?’) as ‘people that seek to improve compassion (as defined in table 1)’, and use the following rules for scoring;

1. App does not mention compassion
2. App offers some relevant information for this target audience but too little to effectively work on improving compassion
3. App offers enough relevant information to effectively work on improving compassion
4. App offers a large amount of relevant information to effectively work on improving compassion but also includes information that is not or less relevant for this target audience.
5. App consists entirely of relevant information to effectively work on improving compassion

- Interpret ‘goals’ in item number 14 (‘does app have specific, measurable and achievable goals (specified in app store description or within the app itself)?**’)** as goals that users might reach by using the app, and score N/A if the description only specifies the goal with which the app was developed (e.g.: ‘this app was developed specifically for the purpose of supporting a participant’s mindfulness based practice’).
- Keep the target audience in mind when scoring item 15 (‘is app content correct, well written, and relevant to the goal/topic of the app?’), and use the following rules for scoring:

1. App does not mention compassion.
2. App mentions compassion but (some of) the information is incorrect.
3. App mentions moderately relevant information for this target audience and the information is correct.
4. App mentions relevant information for this target audience and the information is correct.
5. App mentions highly relevant information for this target audience and the information is correct.

- Keep the target audience in mind when scoring item 16 (“is the extent coverage within the scope of the app; and comprehensive but concise?”) and focus on the following criteria in determining comprehensiveness and conciseness: does the app mention compassion? Is a clear definition of compassion offered? Does the app consist of relevant information entirely or is this mixed with less relevant information for the target audience?
- In scoring item 19 (‘has the app been trialled/tested; must be verified by evidence (in published scientific literature)?’) only count peer reviewed papers, and include expert reviews as well as trials with users.
