# Supplementary material for: Compassion apps for better mental health: qualitative review
Source: BJPsych Open. 2023 Aug 4;9(5):e141. doi: 10.1192/bjo.2023.537 (PMC10486246; doi:10.1192/bjo.2023.537)
Supplement: Supplementary file 1 [file bjosup.zip › S2056472423005379sup002.docx]

| **App** | **Developer** | **Link** | **Language** | **Cost - Basic** | **Cost - Upgrade** | **Store rating** | **Download numbers/**  **Number of ratings** |
| --- | --- | --- | --- | --- | --- | --- | --- |
| **16Guidelines for a happy life** | NutBolt Games | [https://play.google.com/store/apps/details?id=com.app_16g.app.app_16g](https://eur05.safelinks.protection.outlook.com/?url=https%3A%2F%2Fplay.google.com%2Fstore%2Fapps%2Fdetails%3Fid%3Dcom.app_16g.app.app_16g&data=05%7C01%7CE.Feringa-2%40umcutrecht.nl%7Cf3b46dd44ca34dc38dc608db1bcf6257%7Cdcdf4a3dd0c04a6394cf781981249be5%7C0%7C0%7C638134350403736506%7CUnknown%7CTWFpbGZsb3d8eyJWIjoiMC4wLjAwMDAiLCJQIjoiV2luMzIiLCJBTiI6Ik1haWwiLCJXVCI6Mn0%3D%7C3000%7C%7C%7C&sdata=T8lQgxTacEJiaFq5C6BhAujDampeuuOg4G8NuM12BY4%3D&reserved=0) | English | $ 3.99 | $17.99 | - | 1.000+ |
| **Bodi Posi: Daily Habit** | Lumi Studios | [https://play.google.com/store/apps/details?id=com.LumiInteractive.BodyPosiTest.Android](https://eur05.safelinks.protection.outlook.com/?url=https%3A%2F%2Fplay.google.com%2Fstore%2Fapps%2Fdetails%3Fid%3Dcom.LumiInteractive.BodyPosiTest.Android&data=05%7C01%7CE.Feringa-2%40umcutrecht.nl%7Cf3b46dd44ca34dc38dc608db1bcf6257%7Cdcdf4a3dd0c04a6394cf781981249be5%7C0%7C0%7C638134350403736506%7CUnknown%7CTWFpbGZsb3d8eyJWIjoiMC4wLjAwMDAiLCJQIjoiV2luMzIiLCJBTiI6Ik1haWwiLCJXVCI6Mn0%3D%7C3000%7C%7C%7C&sdata=jDt6gg%2FqGN5EpH9PwhX343iTUB8Sdvkzk1Zddl5Gyt0%3D&reserved=0) | English | Free | - | - | 10.000+ downloads |
| **Breathr: Mindful Moments** | BC Children’s Hospital | [https://play.google.com/store/apps/details?id=ca.phsa.breathrapp](https://eur05.safelinks.protection.outlook.com/?url=https%3A%2F%2Fplay.google.com%2Fstore%2Fapps%2Fdetails%3Fid%3Dca.phsa.breathrapp&data=05%7C01%7CE.Feringa-2%40umcutrecht.nl%7Cf3b46dd44ca34dc38dc608db1bcf6257%7Cdcdf4a3dd0c04a6394cf781981249be5%7C0%7C0%7C638134350403736506%7CUnknown%7CTWFpbGZsb3d8eyJWIjoiMC4wLjAwMDAiLCJQIjoiV2luMzIiLCJBTiI6Ik1haWwiLCJXVCI6Mn0%3D%7C3000%7C%7C%7C&sdata=Hmr%2BeLe5CrYkWGNKMmMCZh0vQyMhmBfetxhlIH8TtpM%3D&reserved=0) | English | Free | - | - | 1.000+ downloads |
| **Buddhist Meditations with Vene** | Venerable Namgyel | [https://play.google.com/store/apps/details?id=net.vplay.tibet](https://eur05.safelinks.protection.outlook.com/?url=https%3A%2F%2Fplay.google.com%2Fstore%2Fapps%2Fdetails%3Fid%3Dnet.vplay.tibet&data=05%7C01%7CE.Feringa-2%40umcutrecht.nl%7Cf3b46dd44ca34dc38dc608db1bcf6257%7Cdcdf4a3dd0c04a6394cf781981249be5%7C0%7C0%7C638134350403736506%7CUnknown%7CTWFpbGZsb3d8eyJWIjoiMC4wLjAwMDAiLCJQIjoiV2luMzIiLCJBTiI6Ik1haWwiLCJXVCI6Mn0%3D%7C3000%7C%7C%7C&sdata=0z4U4kM2C60Fl7T6X6k%2BOrXmgRVKJ7R5u1z3rZ82Vos%3D&reserved=0) | English | Free | $10,99 | - | 1.000+ downloads |
| **Centre for Mindfulness Studies** | William Mero | [https://play.google.com/store/apps/details?id=com.mindfulnessstudies.mind2mindbeta](https://eur05.safelinks.protection.outlook.com/?url=https%3A%2F%2Fplay.google.com%2Fstore%2Fapps%2Fdetails%3Fid%3Dcom.mindfulnessstudies.mind2mindbeta&data=05%7C01%7CE.Feringa-2%40umcutrecht.nl%7Cdfacf0a271894869818d08db20e357f6%7Cdcdf4a3dd0c04a6394cf781981249be5%7C0%7C0%7C638139933021208561%7CUnknown%7CTWFpbGZsb3d8eyJWIjoiMC4wLjAwMDAiLCJQIjoiV2luMzIiLCJBTiI6Ik1haWwiLCJXVCI6Mn0%3D%7C3000%7C%7C%7C&sdata=3eHTCpBukEkA1Em%2F2ebD3MyyoPpCJTsx1jbsl%2Bh%2BPpU%3D&reserved=0) | English | Free | - | - | 1.000+ downloads |
| **Compassion Today!** | L Walker | [https://apps.apple.com/nl/app/compassion-today/id871817897](https://eur05.safelinks.protection.outlook.com/?url=https%3A%2F%2Fapps.apple.com%2Fnl%2Fapp%2Fcompassion-today%2Fid871817897&data=05%7C01%7CE.Feringa-2%40umcutrecht.nl%7Cc558c34deac643ed87d808db20e2c3b5%7Cdcdf4a3dd0c04a6394cf781981249be5%7C0%7C0%7C638139930535094756%7CUnknown%7CTWFpbGZsb3d8eyJWIjoiMC4wLjAwMDAiLCJQIjoiV2luMzIiLCJBTiI6Ik1haWwiLCJXVCI6Mn0%3D%7C3000%7C%7C%7C&sdata=QN%2BS%2BIjaSDcr2cDDzV4xkahq%2F450wFQO5BJP0qeg%2FOo%3D&reserved=0) | English | Free | - | - | - |
| **Humanly: A Mental Health Guide** | Justin Wang | [https://apps.apple.com/nl/app/humanly-a-mental-health-guide/id1580501057](https://eur05.safelinks.protection.outlook.com/?url=https%3A%2F%2Fapps.apple.com%2Fnl%2Fapp%2Fhumanly-a-mental-health-guide%2Fid1580501057&data=05%7C01%7CE.Feringa-2%40umcutrecht.nl%7Cc558c34deac643ed87d808db20e2c3b5%7Cdcdf4a3dd0c04a6394cf781981249be5%7C0%7C0%7C638139930535094756%7CUnknown%7CTWFpbGZsb3d8eyJWIjoiMC4wLjAwMDAiLCJQIjoiV2luMzIiLCJBTiI6Ik1haWwiLCJXVCI6Mn0%3D%7C3000%7C%7C%7C&sdata=lJWuyPDY1pJ0WAkMqXV5TYMyN3S9TNKVxoqLZZ6aqls%3D&reserved=0) | English/  Spanish | Free | - | 5 | 1 rating |
| **Imagine Clarity** | Imagine Clarity | [https://play.google.com/store/apps/details?id=com.imagineclarity.clarity](https://eur05.safelinks.protection.outlook.com/?url=https%3A%2F%2Fplay.google.com%2Fstore%2Fapps%2Fdetails%3Fid%3Dcom.imagineclarity.clarity&data=05%7C01%7CE.Feringa-2%40umcutrecht.nl%7Cf3b46dd44ca34dc38dc608db1bcf6257%7Cdcdf4a3dd0c04a6394cf781981249be5%7C0%7C0%7C638134350403736506%7CUnknown%7CTWFpbGZsb3d8eyJWIjoiMC4wLjAwMDAiLCJQIjoiV2luMzIiLCJBTiI6Ik1haWwiLCJXVCI6Mn0%3D%7C3000%7C%7C%7C&sdata=ml9S8euf6k47wdnKguox32e8WyJg1xFJJtWaZIdCJvw%3D&reserved=0) | English/ French | Free | $12,99 a month or $71,99 a year | - | 10.000+ downloads |
| **Insight Timer - Meditatie** | Insight Network Inc | [https://play.google.com/store/apps/details?id=com.spotlightsix.zentimerlite2](https://eur05.safelinks.protection.outlook.com/?url=https%3A%2F%2Fplay.google.com%2Fstore%2Fapps%2Fdetails%3Fid%3Dcom.spotlightsix.zentimerlite2&data=05%7C01%7CE.Feringa-2%40umcutrecht.nl%7Cf3b46dd44ca34dc38dc608db1bcf6257%7Cdcdf4a3dd0c04a6394cf781981249be5%7C0%7C0%7C638134350403736506%7CUnknown%7CTWFpbGZsb3d8eyJWIjoiMC4wLjAwMDAiLCJQIjoiV2luMzIiLCJBTiI6Ik1haWwiLCJXVCI6Mn0%3D%7C3000%7C%7C%7C&sdata=4pnPtc9EVaqHV7HwmfpoOwUmBBTe0719%2Bdo4sRsm2tA%3D&reserved=0) | English/Dutch | Free | $9,99 a month or $59,99 a year | 4,8 | 5.000.000+ downloads/  210.000 ratings |
| **Jon Kabat-Zinn Meditations** | Mindfulness Apps | [https://play.google.com/store/apps/details?id=com.mindfulnessapps.jkz](https://eur05.safelinks.protection.outlook.com/?url=https%3A%2F%2Fplay.google.com%2Fstore%2Fapps%2Fdetails%3Fid%3Dcom.mindfulnessapps.jkz&data=05%7C01%7CE.Feringa-2%40umcutrecht.nl%7Cf3b46dd44ca34dc38dc608db1bcf6257%7Cdcdf4a3dd0c04a6394cf781981249be5%7C0%7C0%7C638134350403736506%7CUnknown%7CTWFpbGZsb3d8eyJWIjoiMC4wLjAwMDAiLCJQIjoiV2luMzIiLCJBTiI6Ik1haWwiLCJXVCI6Mn0%3D%7C3000%7C%7C%7C&sdata=S7BUc1Rk6Z9vUxSheba%2FhwLSHQZEMKgnWGBBOu4AVgo%3D&reserved=0) | English | Free | $2,99 a month or $28.99 a year | - | 10.000+ downloads/ |
| **KritterKneads** | GRID – Gaming Revolution for Inspiring Development | [https://play.google.com/store/apps/details?id=com.GRID.KritterKneads](https://eur05.safelinks.protection.outlook.com/?url=https%3A%2F%2Fplay.google.com%2Fstore%2Fapps%2Fdetails%3Fid%3Dcom.GRID.KritterKneads&data=05%7C01%7CE.Feringa-2%40umcutrecht.nl%7Cf3b46dd44ca34dc38dc608db1bcf6257%7Cdcdf4a3dd0c04a6394cf781981249be5%7C0%7C0%7C638134350403736506%7CUnknown%7CTWFpbGZsb3d8eyJWIjoiMC4wLjAwMDAiLCJQIjoiV2luMzIiLCJBTiI6Ik1haWwiLCJXVCI6Mn0%3D%7C3000%7C%7C%7C&sdata=KGj6C6uiDtK5g26vaTJFbz%2FZnUEaeZw6mapNiG%2FowoA%3D&reserved=0) | English | Free | - | - | 10+ downloads |
| **Meditopia: Meditatie, Slaap** | Meditopia | [https://play.google.com/store/apps/details?id=app.meditasyon](https://eur05.safelinks.protection.outlook.com/?url=https%3A%2F%2Fplay.google.com%2Fstore%2Fapps%2Fdetails%3Fid%3Dapp.meditasyon&data=05%7C01%7CE.Feringa-2%40umcutrecht.nl%7Cf3b46dd44ca34dc38dc608db1bcf6257%7Cdcdf4a3dd0c04a6394cf781981249be5%7C0%7C0%7C638134350403736506%7CUnknown%7CTWFpbGZsb3d8eyJWIjoiMC4wLjAwMDAiLCJQIjoiV2luMzIiLCJBTiI6Ik1haWwiLCJXVCI6Mn0%3D%7C3000%7C%7C%7C&sdata=mUjOonm27f6PeYWXSAbSS4Act5iR%2BgAi5dJ0j3Y6eFo%3D&reserved=0) | English/ Dutch | $49,99 a year | - | 4,2 | 10.000.000+ downloads/  234.000 ratings |
| **Mesmerize – Visual Meditation** | Rockwell Ventures | [https://play.google.com/store/apps/details?id=app.mesmerize](https://eur05.safelinks.protection.outlook.com/?url=https%3A%2F%2Fplay.google.com%2Fstore%2Fapps%2Fdetails%3Fid%3Dapp.mesmerize&data=05%7C01%7CE.Feringa-2%40umcutrecht.nl%7Cf3b46dd44ca34dc38dc608db1bcf6257%7Cdcdf4a3dd0c04a6394cf781981249be5%7C0%7C0%7C638134350403736506%7CUnknown%7CTWFpbGZsb3d8eyJWIjoiMC4wLjAwMDAiLCJQIjoiV2luMzIiLCJBTiI6Ik1haWwiLCJXVCI6Mn0%3D%7C3000%7C%7C%7C&sdata=ZvV0Jf0mBQp8fkSrNfxmUseDRIIi%2Ft07T13H8fsL%2BnI%3D&reserved=0) | English | $12.99 a month or $69.99 a year | - | 2,2 | 100.000+ downloads/ 121.000 ratings |
| **Mindfulness App** | Saake Buwalda | [https://play.google.com/store/apps/details?id=nl.mindfultraining.mindfulness.nlfree](https://eur05.safelinks.protection.outlook.com/?url=https%3A%2F%2Fplay.google.com%2Fstore%2Fapps%2Fdetails%3Fid%3Dnl.mindfultraining.mindfulness.nlfree&data=05%7C01%7CE.Feringa-2%40umcutrecht.nl%7Cf3b46dd44ca34dc38dc608db1bcf6257%7Cdcdf4a3dd0c04a6394cf781981249be5%7C0%7C0%7C638134350403736506%7CUnknown%7CTWFpbGZsb3d8eyJWIjoiMC4wLjAwMDAiLCJQIjoiV2luMzIiLCJBTiI6Ik1haWwiLCJXVCI6Mn0%3D%7C3000%7C%7C%7C&sdata=4U9XoYskz2WoP9JxZm4ykBZ2Gf3GCJZnm6gORcjNric%3D&reserved=0) | Dutch | Free | $4.99 | 3,9 | 10.000+ downloads/  26 ratings |
| **Mindshine: Mental Health Coach** | Mindshine | [https://play.google.com/store/apps/details?id=app.mindshine](https://eur05.safelinks.protection.outlook.com/?url=https%3A%2F%2Fplay.google.com%2Fstore%2Fapps%2Fdetails%3Fid%3Dapp.mindshine&data=05%7C01%7CE.Feringa-2%40umcutrecht.nl%7Cf3b46dd44ca34dc38dc608db1bcf6257%7Cdcdf4a3dd0c04a6394cf781981249be5%7C0%7C0%7C638134350403580263%7CUnknown%7CTWFpbGZsb3d8eyJWIjoiMC4wLjAwMDAiLCJQIjoiV2luMzIiLCJBTiI6Ik1haWwiLCJXVCI6Mn0%3D%7C3000%7C%7C%7C&sdata=vsqnV8LrvPsdCn7SG1W0XjxU1HzRVBZ7rgX59%2FKTQBI%3D&reserved=0) | English | Free |  | 4,7 | 100.000+ downloads/  16.000 reviews |
| **Nan Wu Amitabha** | Fortunate Kidz | [https://play.google.com/store/apps/details?id=com.fortunatekidz.namoamituofo](https://eur05.safelinks.protection.outlook.com/?url=https%3A%2F%2Fplay.google.com%2Fstore%2Fapps%2Fdetails%3Fid%3Dcom.fortunatekidz.namoamituofo&data=05%7C01%7CE.Feringa-2%40umcutrecht.nl%7Cf3b46dd44ca34dc38dc608db1bcf6257%7Cdcdf4a3dd0c04a6394cf781981249be5%7C0%7C0%7C638134350403736506%7CUnknown%7CTWFpbGZsb3d8eyJWIjoiMC4wLjAwMDAiLCJQIjoiV2luMzIiLCJBTiI6Ik1haWwiLCJXVCI6Mn0%3D%7C3000%7C%7C%7C&sdata=Hv0lDliCMwW%2BOeueZEP4sNRovKr5IgN1p0mGZZqkb1I%3D&reserved=0) | English | Free | - | - | 1.000+ downloads |
| **Ommie** | Ommie LLC | [https://apps.apple.com/nl/app/ommie/id1510381143](https://eur05.safelinks.protection.outlook.com/?url=https%3A%2F%2Fapps.apple.com%2Fnl%2Fapp%2Fommie%2Fid1510381143&data=05%7C01%7CE.Feringa-2%40umcutrecht.nl%7Cc558c34deac643ed87d808db20e2c3b5%7Cdcdf4a3dd0c04a6394cf781981249be5%7C0%7C0%7C638139930535094756%7CUnknown%7CTWFpbGZsb3d8eyJWIjoiMC4wLjAwMDAiLCJQIjoiV2luMzIiLCJBTiI6Ik1haWwiLCJXVCI6Mn0%3D%7C3000%7C%7C%7C&sdata=rw%2FmHEotCXpYe9vhfd1H0MxUQ6kL%2FMlUihKyeoEJUaU%3D&reserved=0) | English | Free | - | - | - |
| **Petit BamBou: Meditatie** | FeelVeryBien sarl | [https://play.google.com/store/apps/details?id=com.petitbambou](https://eur05.safelinks.protection.outlook.com/?url=https%3A%2F%2Fplay.google.com%2Fstore%2Fapps%2Fdetails%3Fid%3Dcom.petitbambou&data=05%7C01%7CE.Feringa-2%40umcutrecht.nl%7Cf3b46dd44ca34dc38dc608db1bcf6257%7Cdcdf4a3dd0c04a6394cf781981249be5%7C0%7C0%7C638134350403736506%7CUnknown%7CTWFpbGZsb3d8eyJWIjoiMC4wLjAwMDAiLCJQIjoiV2luMzIiLCJBTiI6Ik1haWwiLCJXVCI6Mn0%3D%7C3000%7C%7C%7C&sdata=cfOgySBd1JnhnKBVi1DUOfUEKNw8a7%2FplHC5nlTzWko%3D&reserved=0) | English, Dutch, Spanish, French, German and Italian | Free | $7.99 a month or $29.99 every 6 months | 4,5 | 5.000.000+ downloads/  126.000 ratings |
| **Piku – Calm Kids** | Kada Designs Ltd | [https://play.google.com/store/apps/details?id=com.kadadesigns.pikuus](https://eur05.safelinks.protection.outlook.com/?url=https%3A%2F%2Fplay.google.com%2Fstore%2Fapps%2Fdetails%3Fid%3Dcom.kadadesigns.pikuus&data=05%7C01%7CE.Feringa-2%40umcutrecht.nl%7Cf3b46dd44ca34dc38dc608db1bcf6257%7Cdcdf4a3dd0c04a6394cf781981249be5%7C0%7C0%7C638134350403580263%7CUnknown%7CTWFpbGZsb3d8eyJWIjoiMC4wLjAwMDAiLCJQIjoiV2luMzIiLCJBTiI6Ik1haWwiLCJXVCI6Mn0%3D%7C3000%7C%7C%7C&sdata=n8thZfs4M2qZpymp5UnbOD4aRsuX6CApka5U040Y%2FIM%3D&reserved=0) | English | Free | $5.49 a month or $42.99 a year | 4,0 | 10.000+ downloads |
| **Samten: Meditation & Sleep** | Samten Limited | [https://apps.apple.com/nl/app/samten-meditation-sleep/id1332503694](https://eur05.safelinks.protection.outlook.com/?url=https%3A%2F%2Fapps.apple.com%2Fnl%2Fapp%2Fsamten-meditation-sleep%2Fid1332503694&data=05%7C01%7CE.Feringa-2%40umcutrecht.nl%7Cc558c34deac643ed87d808db20e2c3b5%7Cdcdf4a3dd0c04a6394cf781981249be5%7C0%7C0%7C638139930535094756%7CUnknown%7CTWFpbGZsb3d8eyJWIjoiMC4wLjAwMDAiLCJQIjoiV2luMzIiLCJBTiI6Ik1haWwiLCJXVCI6Mn0%3D%7C3000%7C%7C%7C&sdata=FnNVicvIEJndiLCbcVXz4dcW5Gxsw1INso3TYq2Gsq4%3D&reserved=0) | English | Free |  | - | - |
| **The Self Compassion App** | PSYT | [https://apps.apple.com/nl/app/the-self-compassion-app/id1553464180](https://eur05.safelinks.protection.outlook.com/?url=https%3A%2F%2Fapps.apple.com%2Fnl%2Fapp%2Fthe-self-compassion-app%2Fid1553464180&data=05%7C01%7CE.Feringa-2%40umcutrecht.nl%7Cc558c34deac643ed87d808db20e2c3b5%7Cdcdf4a3dd0c04a6394cf781981249be5%7C0%7C0%7C638139930535094756%7CUnknown%7CTWFpbGZsb3d8eyJWIjoiMC4wLjAwMDAiLCJQIjoiV2luMzIiLCJBTiI6Ik1haWwiLCJXVCI6Mn0%3D%7C3000%7C%7C%7C&sdata=1g%2B5IAltGhvYikh3ILV%2FC5TUclAjnvlCAekdPTHVTS0%3D&reserved=0) | English | Free | $57,99 | - | - |
| **Three Good Things App** | Oatmeal Apps | [https://play.google.com/store/apps/details?id=co.plumstudio.threegoodthings](https://eur05.safelinks.protection.outlook.com/?url=https%3A%2F%2Fplay.google.com%2Fstore%2Fapps%2Fdetails%3Fid%3Dco.plumstudio.threegoodthings&data=05%7C01%7CE.Feringa-2%40umcutrecht.nl%7Cf3b46dd44ca34dc38dc608db1bcf6257%7Cdcdf4a3dd0c04a6394cf781981249be5%7C0%7C0%7C638134350403736506%7CUnknown%7CTWFpbGZsb3d8eyJWIjoiMC4wLjAwMDAiLCJQIjoiV2luMzIiLCJBTiI6Ik1haWwiLCJXVCI6Mn0%3D%7C3000%7C%7C%7C&sdata=c%2FkHfZyAW02NfoDE3qdlFzA%2Bkf0FM3mVob%2FdwvNvtMM%3D&reserved=0) | English | Free | $11.99 a year | 4,8 | 100.000+ downloads |
| **Unlock Your Potential - Sleep** | Hypnosis and Meditation for Success, LLC | [https://apps.apple.com/nl/app/unlock-your-potential-sleep/id1124189069](https://eur05.safelinks.protection.outlook.com/?url=https%3A%2F%2Fapps.apple.com%2Fnl%2Fapp%2Funlock-your-potential-sleep%2Fid1124189069&data=05%7C01%7CE.Feringa-2%40umcutrecht.nl%7Cc558c34deac643ed87d808db20e2c3b5%7Cdcdf4a3dd0c04a6394cf781981249be5%7C0%7C0%7C638139930535094756%7CUnknown%7CTWFpbGZsb3d8eyJWIjoiMC4wLjAwMDAiLCJQIjoiV2luMzIiLCJBTiI6Ik1haWwiLCJXVCI6Mn0%3D%7C3000%7C%7C%7C&sdata=nd5HbLIzdXJNxixYRCpvr19aeAMR72m6p1W36%2BDYqOc%3D&reserved=0) | English | $ 3.99 | $17.99 | 4,8 |  |
| **Yoga For Kids – Kids Fitness** | NutBolt Games | [https://play.google.com/store/apps/details?id=com.yogakids.YogaForKidsFitness](https://eur05.safelinks.protection.outlook.com/?url=https%3A%2F%2Fplay.google.com%2Fstore%2Fapps%2Fdetails%3Fid%3Dcom.yogakids.YogaForKidsFitness&data=05%7C01%7CE.Feringa-2%40umcutrecht.nl%7Cf3b46dd44ca34dc38dc608db1bcf6257%7Cdcdf4a3dd0c04a6394cf781981249be5%7C0%7C0%7C638134350403580263%7CUnknown%7CTWFpbGZsb3d8eyJWIjoiMC4wLjAwMDAiLCJQIjoiV2luMzIiLCJBTiI6Ik1haWwiLCJXVCI6Mn0%3D%7C3000%7C%7C%7C&sdata=N0PFI9vPtrwM1nMc%2ByNpYNfxC1NU51Pe5%2BDltX5DH4M%3D&reserved=0) | English | Free | - | - | 100.000+ downloads |
